# Hassall’s Corpuscles as Dynamic and Functionally Specialized Structures

**DOI:** 10.3390/ijms27219456

**Published:** 2026-10-23

**Authors:** Chikako Odaka, Elin Hub, Antal Rot

**Affiliations:** Centre for Microvascular Research, The William Harvey Research Institute, Queen Mary University of London, London EC1M 6BQ, UK

**Keywords:** thymus, thymus epithelial cells, Hassall’s corpuscles, dynamics of Hassall’s corpuscle

## Abstract

Hassall’s corpuscles were first described in 1846 by the British physician and anatomist Arthur Hill Hassall. During histological observations of the human thymus, he discovered distinctive concentric epithelial structures within the medulla, which he termed “Hassall’s corpuscles”. Hassall’s corpuscles are also called Hassall’s bodies. These structures share morphological and molecular features with the epidermis. Since Hassall’s corpuscles express late keratinization markers such as involucrin, keratin 10, and filaggrin, they are considered to be composed of terminally differentiated medullary thymic epithelial cells (mTECs). Recent fate-mapping and single-cell transcriptomic analyses have demonstrated that Hassall’s corpuscles originate from post-Aire mTECs, and their differentiation has been suggested to be Aire-dependent. Hassall’s corpuscles are now recognized as heterogeneous structures that play a role in central tolerance and regulatory T cell (Treg) development. In this review, we summarize recent advances in our understanding of the development of Hassall’s corpuscles within the mTEC lineage. We further describe that Hassall’s corpuscles undergo dynamic changes in size and number during thymic involution and regeneration. Thus, we propose that Hassall’s corpuscles should no longer be regarded as merely regressive structures but rather as functionally active bodies with critical immunological roles.

## 1. Introduction

The thymus plays a central role in establishing a functional immune system, serving as the primary site of T lymphocyte differentiation [1]. Morphologically, the thymus is broadly divided into two compartments: the cortex and the medulla. In addition to developing T cells, these compartments contain diverse cellular populations, including thymic epithelial cells, mesenchymal cells, endothelial cells, and dendritic cells.

Hassall’s corpuscles were first described by the British physician and anatomist Arthur Hill Hassall in *The Microscopic Anatomy of the Human Body in Health and Disease*, published in 1846 [2]. He observed concentric, layered epithelial structures within the thymic medulla and termed them “corpuscles” [2]. Since his initial description, research on thymic morphogenesis and histogenesis has greatly advanced. Hassall’s corpuscles have been identified in several mammalian species; they are well developed in humans and guinea pigs [3], but are extremely small in mice and rats [4], which often makes them difficult to detect without immunostaining. The reason why Hassall’s corpuscles are rare in small rodents remains an open question. In humans, Hassall’s corpuscles are already present by 14–16 weeks of gestation and subsequently increase in both size and number as the thymus develops [5,6,7].

Classic histological studies have described Hassall’s corpuscles in humans as “onion-like” structures whose size and number vary, ranging from 20 μm to over 100 μm in diameter, often exhibiting degenerative changes at their center. In the human thymus, Hassall’s corpuscles display diverse morphological features, including cystic transformation, calcification, necrosis, and other degenerative changes. Raica et al. classified them into four types: juvenile, immature, mature, and senescent. These types are based on histological evaluation [8]. Liberti et al. categorized them into solid and cystic types, based on the presence or absence of internal voids within the corpuscles [9].

In this review, we provide an overview of Hassall’s corpuscles and summarize the current understanding of their immunological significance. We also highlight recent advances suggesting that the diverse cell aggregates, through complex cellular interactions, are involved in efficient thymic function.

## 2. Hassall’s Corpuscles

### 2.1. Molecular Markers and Cellular Composition

Hassall’s corpuscles in humans exhibit morphological features similar to those of epidermal cells. Ultrastructural and histochemical analyses have revealed comparable patterns of protein expression between Hassall’s corpuscles and the epidermis [10]. As summarized in Table 1, several proteins have been reported as characteristic markers of Hassall’s corpuscles.

They display a phenotype reminiscent of keratinized epithelium (stratum corneum), characterized by a strong expression of late keratinocyte differentiation markers such as involucrin [11], keratin 1/10 [12,13], and filaggrin [14], often accompanied by nuclear loss. The α3 subunit of the nicotinic acetylcholine receptor is expressed in terminally differentiated epithelial cells in both the thymic medulla and skin [15], although its function remains unclear. Tight junction proteins claudin-1 and claudin-4 are expressed in some mTECs near Hassall’s corpuscles, facilitating cohesion among keratinized epithelial cells and maintaining their structure [16]. Thus, in addition to their distinctive histological appearance, Hassall’s corpuscles express antigens typically found in the terminally differentiated upper layers of the epidermis, supporting the view that they are composed of terminally differentiated mTECs.

Hassall’s corpuscles also express ligands for *Tetragonolobus purpureus* agglutinin (TPA), also known as *Lotus tetragonolobus* lectin (LTA) [17]. TPA binds strongly to glycans containing α1,3-linked fucose residues, particularly Lewis x antigens [18,19]. Stage-specific embryonic antigen-1 (SSEA-1), also known as Lewis x antigen and CD15, is a well-established marker for mouse embryonic stem cells (ES cells) [20,21]. This antigen is present on undifferentiated ES cells but absent from differentiated progeny [22,23]. Therefore, SSEA-1 is a useful marker for tracking the degree of stem cell differentiation, and its expression has come to be regarded as a useful indicator of the undifferentiated state not only in ES cells but also in other stem cell types. For example, SSEA-1 is abundantly expressed in neural stem cells of both fetal and adult mouse and human brains, supporting the concept that SSEA-1 expression marks the undifferentiated state of stem cells beyond ES cells [24]. It is conceivable that TPA recognizes SSEA-1 expressed in Hassall’s corpuscles. Because most commercially available antibodies against SSEA-1 are mouse monoclonal antibodies, and given that immunoglobulins are present within Hassall’s corpuscles [25], appropriate procedures are required for immunohistochemical analyses in mouse tissues.

Hassall’s corpuscles are not simply aggregates of epithelial cells but are heterogeneous structures that frequently contain non-epithelial cells [26]. Indeed, most of Hassall’s corpuscles are composed of thymic epithelial cells, macrophages, infiltrating dendritic cells (DCs), myoid cells, and occasionally mast cells or lymphocytes. During their turnover, enlarged Hassall’s corpuscles are phagocytosed and removed by macrophages. In the aged human thymus, macrophages within Hassall’s corpuscles express insulin-like growth factor 1 (IGF-1) and its receptor [27]. Moreover, Hassall’s corpuscles have been shown to express several cytokines, including IL-7, CD30 ligand, stromal cell-derived factor 1 (SDF-1), macrophage-derived chemokine, and thymic stromal lymphopoietin (TSLP) [28,29,30].

### 2.2. Immunological Functions

Hassall’s corpuscles were originally thought to be regressive structures, and their functional significance remained unclear for decades. It is now recognized that they act as active immunological units involved in the induction of central tolerance [31]. Hassall’s corpuscles secrete TSLP, which activates CD11c^+^ DCs within the thymus. These TSLP-activated DCs subsequently promote the differentiation of CD4^+^CD8^−^CD25^−^ thymocyte precursors into Foxp3^+^ regulatory T cells (Tregs), thereby demonstrating a key role for Hassall’s corpuscles in supporting Treg development in the thymic microenvironment [31]. Claudin-1 has been shown to recruit DCs to the vicinity of mTECs and anchor them there. In claudin-1-deficient mice, DCs are unable to aggregate near mTECs, leading to a breakdown of their proper positioning within the medulla [32].

The autoimmune regulator (Aire) is an mTEC-specific transcription factor required for the expression of tissue-specific antigens and the induction of central tolerance [33]. In Aire knockout (KO) mice, Hassall’s corpuscle-like structures are reported to be largely absent [34,35]. In Aire KO mice, the absence of Hassall’s corpuscle formation correlates with impaired negative selection of autoreactive T cells and defective Treg differentiation in the thymus, suggesting that Hassall’s corpuscles contribute to maintaining central tolerance through both negative selection and Treg induction.

In addition to their role in T cell tolerance, Hassall’s corpuscles are also associated with humoral immunity. Human thymic tissue has been reported to express multiple immunoglobulin isotypes (IgG, IgA, IgM, IgD, and IgE), as well as light chains, within Hassall’s corpuscle cells [25]. The production of immunoglobulins in the thymus and their localization within Hassall’s corpuscles may contribute to the maturation and regulation of immune responses.

### 2.3. Developmental Origin

**Figure 1:**
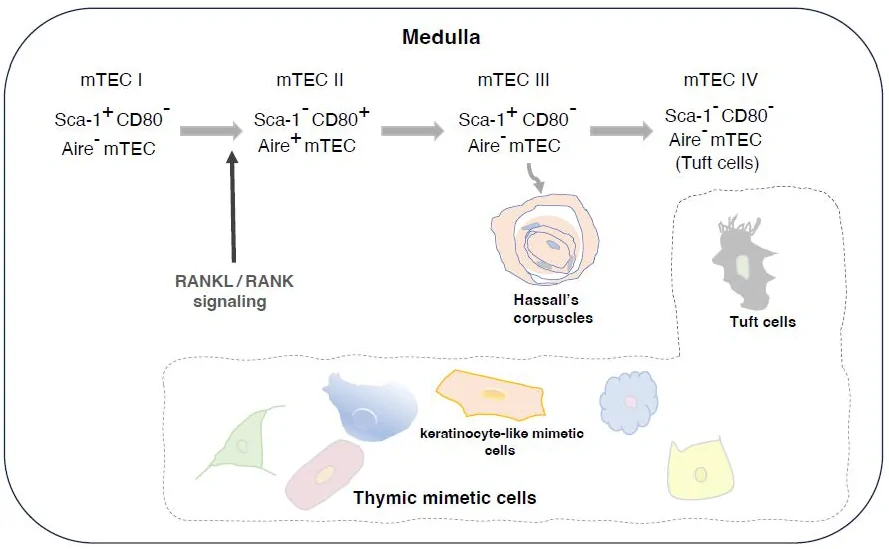
A schematic representation of the proposed lineage among mTEC subsets. Advances in single-cell RNA sequencing (scRNA-seq) have uncovered a previously unappreciated degree of TEC heterogeneity [40]. Hassall’s corpuscles arise from post-Aire mTECs (mTEC III). mTEC IV/thymic tuft cells originate from mTEC III and, in part, from mTEC II. Aire expression is regulated by RANKL–RANK signaling. With recent progress in comprehensive, high-resolution single-cell and chromatin accessibility analyses [42], it has become clear that mimetic cells, except thymic tuft cells, cannot be neatly categorized into mTEC I–IV subsets. Instead, they constitute distinct transcription factor-driven populations that branch out radially and diversely from the mature mTEC pool.

The development of Hassall’s corpuscles parallels the differentiation of Aire^+^ mTECs, suggesting that they represent a post-Aire cellular population. Recent fate-mapping and single-cell transcriptomic analyses have revealed previously unrecognized heterogeneity among TECs [36,37,38,39,40]. These analyses demonstrated that Hassall’s corpuscles arise from post-Aire medullary thymic epithelial cells (mTEC III), which have downregulated Aire expression and subsequently undergo terminal keratinocyte-like differentiation (Figure 1). Human post-Aire mTECs and Hassall’s corpuscles produce the endogenous TLR4 agonists S100A8/S100A9, implicating them in the induction of a pro-inflammatory microenvironment [41].

Members of the tumor necrosis factor receptor (TNFR) superfamily, including CD40, LTβR, and receptor activator of nuclear factor κB (RANK), activate the non-canonical nuclear factor κB (NF-κB) signaling pathway [43]. Aire expression is regulated by this atypical NF-κB pathway, which is triggered by RANK signaling [44,45]. Importantly, NF-κB binding sites within a conserved noncoding sequence (CNS1) upstream of the Aire gene are essential for RANK-dependent Aire expression. Deficiency of RANKL–RANK signaling results in profound defects in mTEC development [46,47,48]. Furthermore, mice lacking non-canonical NF-κB signaling components such as NIK, IKKα, or RelB/p52 exhibit aberrant Aire expression, severe defects in mTEC development, and impaired central tolerance [49,50,51,52]. In humans, RelB deficiency leads to thymic dysplasia accompanied by a marked reduction in Hassall’s corpuscles [53].

Beyond regulating Aire, RANK signaling also induces interferon-stimulated genes via a mechanism involving STAT1 phosphorylation [54]. Immunohistochemical analyses have revealed abundant STAT1 activation within Hassall’s corpuscles in the human thymus [55], raising the possibility that RANK signaling drives STAT1 activation in these structures. Moreover, activation of the MAPK and JAK–STAT pathways, similar to those involved in keratinocyte differentiation, appears to play a role in Hassall’s corpuscle formation. However, three MAPK family members, extracellular signal-regulated kinase (ERK), c-Jun N-terminal kinase (JNK), and p38 kinase, exhibit distinct expression patterns within the thymic medulla, including within Hassall’s corpuscles [56,57]. Further investigation is required to clarify the precise roles of these kinases in the development of Hassall’s corpuscles.

### 2.4. Hassall’s Corpuscles and Diseases

Hassall’s corpuscles are known to undergo structural changes with aging. During age-related thymic involution, both their size and number decline in humans and mice [58,59], and these changes may be associated with a decline in thymic function and immunosenescence.

Autoimmune polyglandular syndrome type 1 (APS-1), also known as autoimmune polyendocrinopathy-candidiasis-ectodermal dystrophy (APECED), is caused by mutations in the Aire gene [60,61]. In Aire-deficient mouse models, the absence of Hassall’s corpuscles is associated with defective central tolerance and the development of autoimmunity [62]. In patients with Down syndrome, the presence of an additional copy of chromosome 21 (which harbors the *Aire* gene) leads to an increased frequency of Aire^+^ cells and enlarged Hassall’s corpuscles, suggesting that elevated Aire expression in this condition may influence mTEC maturation [63,64]. In thymic tissues from immunodeficient patients with reduced thymopoiesis, Hassall’s corpuscles are completely absent [65,66,67]. In children with human immunodeficiency virus (HIV) infection, common thymic abnormalities include involution or atrophy, thymitis, and abnormal involution characterized by the loss of Hassall’s corpuscles [68].

On the other hand, thymic hyperplasia is defined by an increase in the number of thymocytes [69]. In myasthenia gravis patients with thymic hyperplasia, the number of Hassall’s corpuscles is also increased [70], and mutation of p53 is detectable even in myasthenia gravis [71]. p53 plays a critical role in regulating apoptosis and is preferentially expressed in normal cTECs [72]. These findings suggest that alterations in Hassall’s corpuscles may contribute to immune dysregulation and disease development.

## 3. Thymic Mimetic Cells

Tuft cells (also known as brush cells) in the intestinal and respiratory tracts are Dclk1^+^TRPM5^+^ chemosensory cells that secrete IL-25 and activate type 2 innate lymphoid cells (ILC2s). TECs expressing tuft cell markers such as Pou2f3, TRPM5, DCLK1, and L1CAM have been identified within or surrounding Hassall’s corpuscles [39,40]. Thymic tuft cells are scarcely detectable during the neonatal period. Compared with peripheral tuft cells, they are distinguished by the expression of L1CAM and relatively high expression of the antigen-presenting molecules MHC class II and CD74 [73]. IL-25 expression in thymic tuft cells promotes IL-4 production by natural killer T type 2 (NKT2) cells, which, in turn, influences the development and immune responses of ILC2s. Thus, thymic tuft cells share features with mucosal tuft cells found in the intestinal and respiratory tracts. mTECs that mimic the phenotype and gene expression profiles of diverse peripheral tissues, such as the skin, gut, lung, and muscle, have been identified and collectively termed “mimetic cells” [42]. mTEC IV/thymic tuft cells originate from post-Aire mTEC (Figure 1). Although thymic tuft cells are localized within or adjacent to Hassall’s corpuscles, their precise relationship with these structures remains unclear.

## 4. Dynamics of Hassall’s Corpuscles

### 4.1. Changes in Hassall’s Corpuscles During Acute Injury/Regeneration in the Thymus

Hassall’s corpuscles have been reported to both increase and decrease under different experimental conditions. In guinea pigs subjected to X-irradiation or cortisone administration, Hassall’s corpuscles exist in a dynamic equilibrium: they increase during acute thymic involution and decrease following regeneration [74,75]. Similar findings have been reported in cats, rabbits, and mice. In mice subjected to total-body irradiation, Hassall’s corpuscles were found to increase in both size and number during acute thymic involution, as shown by immunostaining, suggesting that their abundance reflects thymic activity.

Rode and Boehm established Ccrl1:hDTR transgenic mice (with Ccrl1 being the historical name for ACKR4), enabling selective diphtheria toxin (DT)-mediated ablation of the cTEC compartment [76]. Acute cTEC depletion in mice is accompanied by a dramatic reduction in immature thymocyte populations. Strikingly, we found that acute ablation of cTECs also promotes the development of Hassall’s corpuscles, implying that these structures may play a role in thymic regeneration [77]. Thymic crosstalk, defined as a reciprocal regulatory network between thymocytes and the thymic stroma, is essential for proper thymic architecture [78]. Recent evidence further suggests that such crosstalk also occurs between TEC subsets. Venables et al. demonstrated that cortical epithelial morphology during thymic atrophy and regeneration is regulated by signals from the medullary stroma [79]. The mechanism by which acute cTEC depletion promotes Hassall’s corpuscle development remains unclear; however, mTEC differentiation may be regulated by signals from cTECs and immature thymocytes.

### 4.2. Changes in Hassall’s Corpuscles Associated with Developmentally Driven Alterations in TEC Differentiation

TGF-β is a key regulator of epithelial cell differentiation and stem cell maintenance. Specifically, it suppresses epithelial proliferation and promotes maintenance of an undifferentiated state, thereby regulating tissue homeostasis and regeneration [80]. Although the role of TGF-β signaling in osteoclastogenesis remains controversial, several studies have shown that TGF-β is involved in RANKL-induced osteoclastogenesis and TGF-β signaling suppresses RANKL-induced osteoclast differentiation [81]. In TGFβRII ^lox/lox^::Foxn1-Cre mice, in which TGFβRII is specifically deleted in Foxn1-expressing cells, age-related thymic involution was alleviated, and early reconstitution after lethal irradiation was observed [82]. Prominent formation of Hassall’s corpuscles was also detected in these mice [83], suggesting that TGF-β signaling inhibits the development of Hassall’s corpuscles and may also regulate RANKL-induced mTEC differentiation toward the terminal stage. Together, these findings suggest that increases in the size and number of Hassall’s corpuscles correlate with thymopoietic activity.

BRCA1 (breast cancer 1), a tumor suppressor associated with hereditary breast and ovarian cancer, is highly expressed in the thymus, though its physiological role in the thymus remains unclear. BRCA1 was found to be expressed in a subset of mTECs characterized as EpCAM (CD326)^+^, UEA-1 ligand^+^, and Aire^−^ [84]. To clarify its functional significance, we analyzed TEC differentiation in K14cre;Brca1F/F mice, in which BRCA1 is specifically deleted in K14-expressing cells [84]. Interestingly, conditional BRCA1 deficiency resulted in enhanced development of Hassall’s corpuscles. There are structural differences between cTECs and mTECs that are closely related to their immunological roles within the thymic microenvironment. cTECs display a complex stellate morphology with elongated cytoplasmic projections forming a three-dimensional meshwork, a small nucleus, and abundant lysosomes and endosomes, reflecting their active role in antigen processing. In contrast, mTECs are generally larger, more rounded, and form denser clusters. Notably, in BRCA1 knockout mice, β5t^+^ cells were frequently observed adjacent to Hassall’s corpuscles, and some β5t^+^ cells formed small spherical structures in the medulla. Some medullary β5t^+^ cells expressed Ly-51, and CD205 expression was detected in these β5t^+^ cells at the corticomedullary junction. We hypothesized that Hassall’s corpuscles may serve as “cradles” from which cTECs emerged and migrated toward the cortical region in K14cre;Brca1F/F adult mice.

Although the relationship between BRCA1 and Foxn1 remains unclear, these findings suggest that the development of Hassall’s corpuscles may promote the emergence of β5t^+^ cTEC progenitors, or alternatively, that these progenitors may originate from Hassall’s corpuscles. Further studies are needed to clarify how Hassall’s corpuscles may generate a niche for Foxn1^+^β5t^+^ cTEC progenitors.

All findings described in this section of dynamics of Hassall’s corpuscles were obtained in guinea pigs, cats, rabbits, or mice.

## 5. Recent Progress

Advanced three-dimensional X-ray imaging has revealed the structural development of human thymic compartments, demonstrating that Hassall’s corpuscles occupy approximately 25% of the medullary volume by four months after birth [85]. This observation suggests that Hassall’s corpuscles can be regarded as a distinct compartment of the thymus and may play a role as a quantitatively prominent component of thymic architecture.

Recent studies have also highlighted the roles of thymic tuft cells and fibroblasts in thymic regeneration via the activation of type 2 immune responses in mice [86]. ILC2 cells promote tissue regeneration by producing effector molecules, thereby facilitating mTEC differentiation following acute thymic involution. The dynamic behavior of Hassall’s corpuscles is also associated with thymic regeneration following acute involution. Investigating the interplay between thymic tuft cells and Hassall’s corpuscles is therefore of considerable interest for elucidating the mechanisms underlying thymic maintenance and recovery.

## 6. Conclusions

The remarkable heterogeneity of TECs, revealed by single-cell RNA sequencing, provides a partial explanation for the complexity and functional importance of these structures in thymic physiology. Further single-cell RNA-seq analyses of mTECs will likely advance our understanding of the development, organization, and immunological functions of Hassall’s corpuscles and clarify their contributions to immune tolerance. Future studies are expected to elucidate the mechanisms underlying Hassall’s corpuscle formation following acute thymic involution, with particular emphasis on the differentiation pathways from mTEC III and the involvement of specific transcription factors. Although innate immune components are known to influence thymic regeneration, elucidating how the innate immune system affects precursor TECs and/or their differentiated progeny may provide novel therapeutic targets for modulating thymic function.

In summary, Hassall’s corpuscles are increasingly being redefined from mere terminally differentiated structures to multifunctional immunoregulatory units within the thymic medulla, and integrated analyses combining cellular, molecular, and spatial information are expected to provide new insights into thymic biology and central tolerance, as well as to facilitate the development of potential regenerative therapies.

## Figures and Tables

**Table 1:** Molecular markers of Hassall’s corpuscles.

Protein	References	Species	Cellular Localization
involucrin	[11]	human/mouse	cytoplasm
keratin 1/10	[12,13]	human/mouse	cytoplasm
filaggrin	[14]	human	cytoplasm
The α3 subunit of the nicotinic acetylcholine receptor	[15]	human	cell surface
a ligand for Tetragonolobus purpureus agglutinin (TPA), also known as Lotus tetragonolobus lectin (LTA)	[16]	mouse	cell surface

## Data Availability

No new data were created or analyzed in this study. Data sharing is not applicable to this article.

## Abbreviations

TECs, thymic epithelial cells; cTECs, cortical thymic epithelial cells; mTECs, medullary thymic epithelial cells; CMJ, corticomedullary junction; Tregs, regulatory T cells; TPA, Tetragonolobus purpureus agglutinin; LTA, Lotus tetragonolobus; SSEA-1, stage-specific embryonic antigen; TSLP, thymic stromal lymphopoietin; DCs, dendritic cells; Aire, autoimmune regulator; UEA-1, Ulex europaeus agglutinin-1.
